# Naturally Drug-Loaded Chitin: Isolation and Applications

**DOI:** 10.3390/md17100574

**Published:** 2019-10-10

**Authors:** Valentine Kovalchuk, Alona Voronkina, Björn Binnewerg, Mario Schubert, Liubov Muzychka, Marcin Wysokowski, Mikhail V. Tsurkan, Nicole Bechmann, Iaroslav Petrenko, Andriy Fursov, Rajko Martinovic, Viatcheslav N. Ivanenko, Jane Fromont, Oleg B. Smolii, Yvonne Joseph, Marco Giovine, Dirk Erpenbeck, Michael Gelinsky, Armin Springer, Kaomei Guan, Stefan R. Bornstein, Hermann Ehrlich

**Affiliations:** 1 Department of Microbiology, National Pirogov Memorial Medical University, Vinnytsia 21018, Ukraine; valentinkovalchuk2015@gmail.com; 2 Department of Pharmacy, National Pirogov Memorial Medical University, Vinnytsia 21018, Ukraine; algol2808@gmail.com; 3 Institute of Pharmacology and Toxicology, TU Dresden, Dresden 01307, Germany; Bjoern.Binnewerg@tu-dresden.de (B.B.); mario.schubert1@tu-dresden.de (M.S.); Kaomei.Guan@tu-dresden.de (K.G.); 4 V.P. Kukhar Institute of Bioorganic Chemistry and Petrochemistry, National Academy of Science of Ukraine, Murmanska Str. 1, Kyiv 02094, Ukraine; lmuzychka@rambler.ru (L.M.); smolii@bpci.kiev.ua (O.B.S.); 5 Institute of Chemical Technology and Engineering, Faculty of Chemical Technology, Poznan University of Technology, Berdychowo 4, Poznan 60965, Poland; 6 Institute of Electronic and Sensor Materials, TU Bergakademie Freiberg, Gustav-Zeuner Str. 3, Freiberg 09599, Germany; iaroslavpetrenko@gmail.com (I.P.); andriyfur@gmail.com (A.F.); Yvonne.Joseph@esm.tu-freiberg.de (Y.J.); 7 Leibniz Institute for Polymer Research Dresden, Dresden 01069, Germany; tsurkan@ipfdd.de; 8 Institute of Clinical Chemistry and Laboratory Medicine, University Hospital Carl Gustav Carus, Faculty of Medicine Carl Gustav Carus, TU Dresden, Dresden 01307, Germany; Nicole.Bechmann@uniklinikum-dresden.de; 9 Institute of Marine Biology, University of Montenegro, Kotor 85330, Montenegro; rajko.mar@ucg.ac.me; 10 Department of Invertebrate Zoology, Biological Faculty, Lomonosov Moscow State University, Moscow 119992, Russia; ivanenko.slava@gmail.com; 11 Aquatic Zoology Department, Western Australian Museum, Locked Bag 49, Welshpool DC, Western Australia WA6986, Australia; Jane.Fromont@museum.wa.gov.au; 12 Department of Sciences of Earth, Environment and Life, University of Genoa, Corso Europa 26, 16132 Genova, Italy; mgiovine@unige.it; 13 Department of Earth and Environmental Sciences & GeoBio-Center, Ludwig-Maximilians-Universität München, Richard-Wagner-Str. 10, Munich 80333, Germany; erpenbeck@lmu.de; 14 Centre for Translational Bone, Joint and Soft Tissue Research, Faculty of Medicine and University Hospital Carl Gustav Carus of Technische Universität Dresden, Fetscherstraße 74, Dresden 01307, Germany; michael.gelinsky@tu-dresden.de (M.G.); Armin.Springer@med.uni-rostock.de (A.S.); 15 Medizinische Biologie und Elektronenmikroskopisches Zentrum (EMZ), Universitätsmedizin Rostock, Rostock 18055, Germany; 16 Department of Internal Medicine III, University Hospital Carl Gustav Carus, Technische Universität Dresden, Dresden 01307, Germany; Stefan.Bornstein@uniklinikum-dresden.de; 17 Diabetes and Nutritional Sciences Division, King’s College London, London WC2R 2LS, UK

**Keywords:** chitin, scaffolds, pigmental cells, demosponges, Ianthella, bromotyrosines, decamethoxine, drug delivery

## Abstract

Naturally occurring three-dimensional (3D) biopolymer-based matrices that can be used in different biomedical applications are sustainable alternatives to various artificial 3D materials. For this purpose, chitin-based structures from marine sponges are very promising substitutes. Marine sponges from the order Verongiida (class Demospongiae) are typical examples of demosponges with well-developed chitinous skeletons. In particular, species belonging to the family Ianthellidae possess chitinous, flat, fan-like fibrous skeletons with a unique, microporous 3D architecture that makes them particularly interesting for applications. In this work, we focus our attention on the demosponge *Ianthella flabelliformis* (Linnaeus, 1759) for simultaneous extraction of both naturally occurring (“ready-to-use”) chitin scaffolds, and biologically active bromotyrosines which are recognized as potential antibiotic, antitumor, and marine antifouling substances. We show that selected bromotyrosines are located within pigmental cells which, however, are localized within chitinous skeletal fibers of *I. flabelliformis*. A two-step reaction provides two products: treatment with methanol extracts the bromotyrosine compounds bastadin 25 and araplysillin-I N20 sulfamate, and a subsequent treatment with acetic acid and sodium hydroxide exposes the 3D chitinous scaffold. This scaffold is a mesh-like structure, which retains its capillary network, and its use as a potential drug delivery biomaterial was examined for the first time. The results demonstrate that sponge-derived chitin scaffolds, impregnated with decamethoxine, effectively inhibit growth of the human pathogen *Staphylococcus aureus* in an agar diffusion assay.

## 1. Introduction

**Figure 1:**
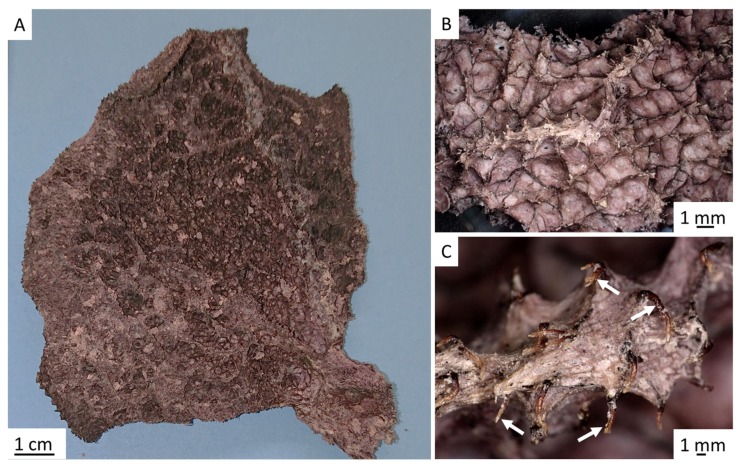
The marine demosponge *Ianthella flabelliformis* (Linnaeus, 1759) (**A**), as collected after air-drying, exhibits a characteristic fan-like and meshwork morphology (**B**). Chitin-based skeletal fibers (arrows) are visible between tissue-like layers (**C**).

**Figure 2:**
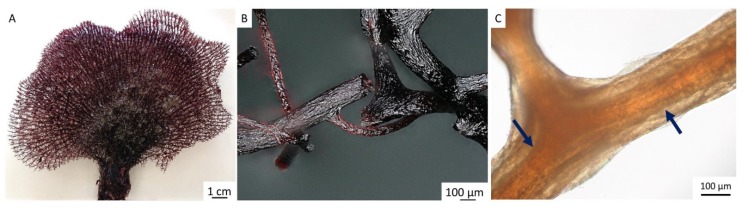
The cell-free macerated skeleton of *I. flabelliformis* (**A**,**B**) is made of anastomosing, interconnected tube-based chitinous fibers (**B**). Partial depigmentation using alkali treatment leads to visualization of the inner channel (arrows, **C**) which is located within each fiber.

Development of three-dimensional (3D) scaffolds based on natural biopolymers is a recent trend in materials and biomaterials science. The scientific community now focuses on development of fabrication methods which will allow for precise control of the architecture and pore structures in such scaffolds. Evolutionary optimized 3D constructs of natural origin can be found in marine demosponges (phylum Porifera, class Demospongiae), which are recognized among the first multicellular organisms on our planet. These organisms evolved and survived for more than 600 million years due to their ability to excellently combine the mechanical stability of their voluminous, fiber-based, water-filtering skeletons [1] and their diverse chemical defense strategies [2,3] due to biosynthesis of secondary metabolites with anti-predatory and antibiotic properties [4]. The demosponges can produce both proteinaceous (spongin)- or polysaccharide (chitin)-based and up to 2-m-high skeletons (for an overview, see References [5,6,7,8,9,10]). Representatives of the family Ianthellidae (Hyatt, 1875) possess chitinous, flat, fan-like skeletons with unique 3D architecture [11,12] (Figure 1 and Figure 2A), some of which can reach up to 2 m in diameter [12].

One main advantage of skeletons of the ianthellid sponges with respect to their practical application is that chitinous scaffolds can be easily isolated from them (for details, see Reference [11]). These scaffolds exhibit the characteristic shape, size, and meshwork-like structural motif of demosponges, throughout which mesophyll cells are perfectly distributed (Figure 1B,C). Recently, 3D chitin scaffolds isolated from the demosponge *Ianthella basta* (Pallas, 1766) were successfully used in tissue engineering of human mesenchymal stromal cells (hMSC), as well as human dermal MSCs [13]. Additional advantages of these sponges for use in technological [14,15] and biomedical applications [16] is their exceptional ability to regenerate their skeletons in situ, with a growth rate of up to 12 cm/year [12]. Consequently, marine farming of these sponges in coastal areas in Australia and Guam (United States of America) [17] is planned to be developed in the near future.

Currently, our strategy regarding the ianthellid sponges is focused on development of methods for simultaneous extraction of both naturally pre-designed chitin (as “ready-to-use” scaffolds) and biologically active bromotyrosines which are recognized as potential antibiotic, antitumor (for a review, see References [18,19]), and marine antifouling [20,21] substances. The pharmacological potential of these marine demosponges as producers of bromotyrosine-related bastadins was very positively approved [22,23,24,25,26,27].

In this work, we focus our attention on *Ianthella flabelliformis* (Linnaeus, 1759) (Demospongiae, Verongiida, Ianthellidae) [28], originally designated by Linneaus as *Spongia flabelliformis* [29] and later transferred to the genus *Ianthella* (Gray, 1869), for simultaneous extraction of both naturally occurring chitin scaffolds, and biologically active bromotyrosines which are recognized as potential antibiotic, antitumor, and marine antifouling substances. In this species, bromotyrosines are located within specialized, pigmental cells that are tightly associated with skeletal chitin. Special attention was paid to investigations on the applicability of 3D chitinous spacer fabric-like scaffolds as potential drug delivery biomaterials, filled with a quaternary ammonium compound, decamethoxine [30], which is a well-recognized antiseptic against diverse human diseases [31]. We used a clinical strain of the human pathogen *Staphylococcus aureus* as the test microorganism in this study. 

## 2. Results

The methodology of how to obtain chitin in the size, shape, and unique architecture of the *I. flabelliformis* fan-like skeleton is described in Section 4.2. However, one of the most important steps is obtaining a pigmented skeletal structure that is tissue-free (Figure 2A,B). Additional treatment with 5% NaOH at 37 °C over 4 h exposes chitinous fibers with visible inner channels which are typically located within skeletal fibers of verongiid demosponges (see also References [9,11]). These channels permit verongiid chitin to be saturated with diverse liquids due to the capillary effect (for details, see Reference [32]), and the presence of similar channels in our samples suggests that a similar mechanism of capillary uptake might be possible in the skeleton of *I. flabelliformis* (Figure 2C). 

**Figure 3:**
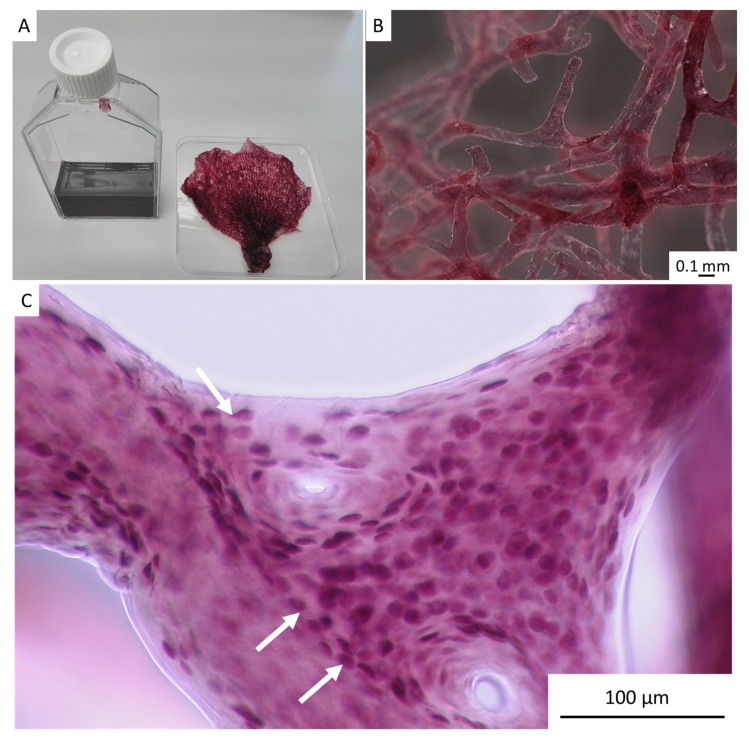
The bromotyrosine-containing extract isolated from skeleton of *I. flabelliformis* (**A**) is one of the sources of pharmacologically relevant reagents. The dark-reddish color of chitinous skeletal fibers (**B**) is determined by the presence of pigmental cells, or spherulocites (**C**), special chitin-associated bromotyrosine-producing cells (arrows).

Importantly, using this method, isolation of pigmented chitinous fibers (Figure 3) is possible, in which the presence of reddish-violet cells was visualized using light microscopy (Figure 3C). These so-called “fiber cells” [33], “*pigmental cells*” [34,35,36,37], or “*spherulous cells*” [38,39] are presumable characteristic features for representatives of the Ianthellidae family [37,40]. The size of these cells in *I. flabelliformis* is 11.8 µm ± 1.15 µm.

**Figure 4:**
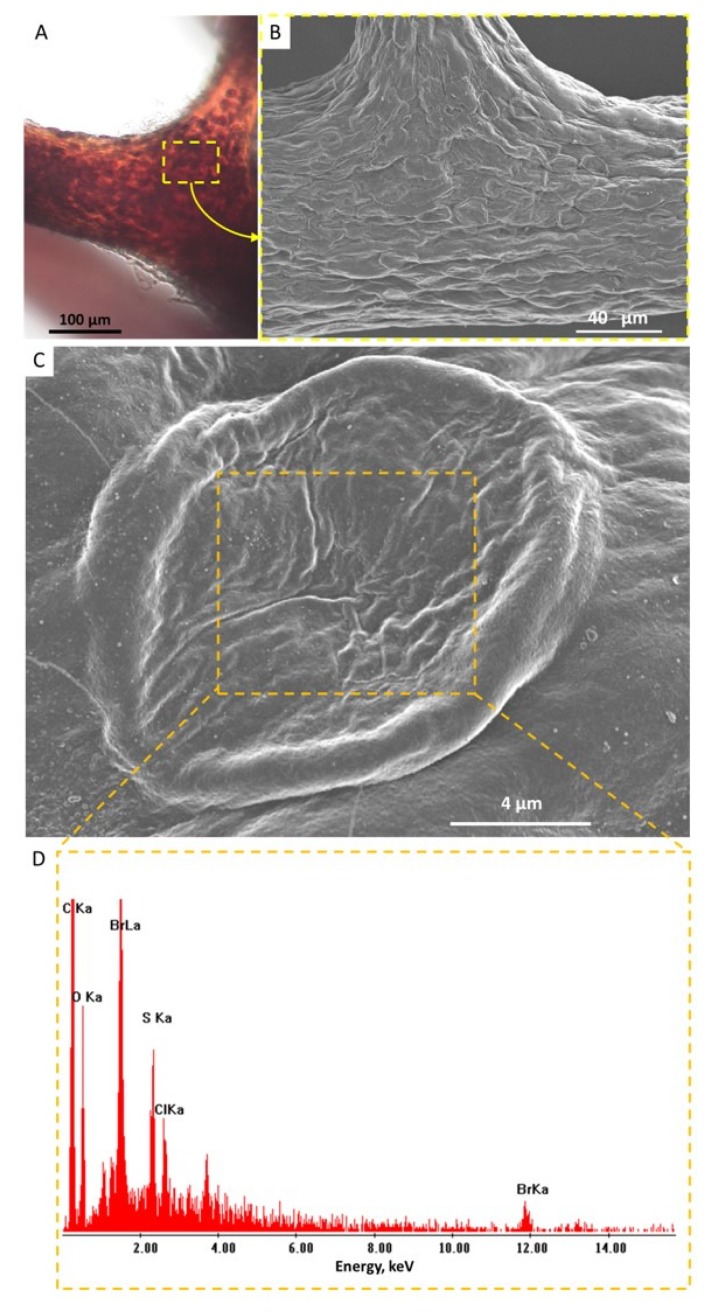
Pigmental cells located within fibers of *I. flabelliformis* chitin are clearly visible using light microscopy (**A**) (see also Figure 3C). These cells are observable using SEM (**B**,**C**). Single-spot energy-dispersive X-ray spectroscopy (EDX) analysis shows strong evidence of the presence of bromine within individual pigmental cells (**D**).

Single-spot energy-dispersive X-ray spectroscopy (EDX) analysis using SEM strongly confirmed the presence of bromine within individual pigmental cells (Figure 4). These results correlate well with those reported previously for similar cells observed in the verongiid demosponge *Aplysina aerophoba* (Nardo, 1833) [39]. 

**Figure 5:**
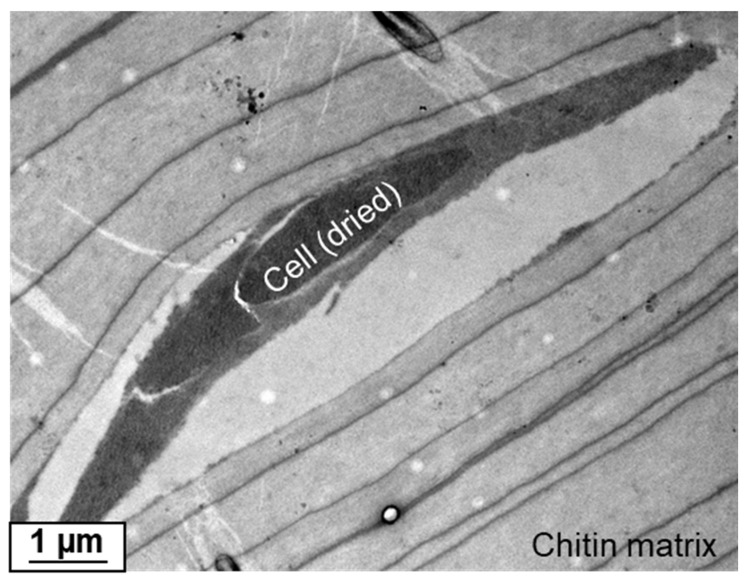
TEM image of the cross-section through the chitinous sponge matrix of *I. flabelliformis* showing the interlayer location of the pigmental cell that lost its oval morphology due to the drying procedure. This cell is definitively of eukaryotic and not bacterial origin.

TEM analysis of the cross-sectioned chitinous fiber of *I. flabelliformis* studied during the isolation step, as represented in Figure 3C and Figure 4A, showed the location of such cells between the alternating chitinous layers (Figure 5). 

Due to the evident presence of bromine within pigmented cells of *I. flabelliformis*, we suggest that bromotyrosines are responsible for this naturally occurring event. Thus, the corresponding methanol extract obtained after treatment of the pigmented skeletal fibers (Figure 3) was subjected to HPLC for isolation, analysis, and identification of possible bromotyrosine derivatives. Compounds were identified by analyzing the mass spectra and comparing with the data obtained for the same compounds previously isolated from *I. flabelliformis* sponge [41,42]. Our data (see Supplementary Materials) indicate that the isolated compounds correspond to bastadin 25 (MS (electrospray ionization, ESI): *m*/*z* 1032 [M − H]^−^) [41] and araplysillin-I *N*^20^-sulfamate (MS (ESI): *m*/*z* 794 [M − Na]^−^) [42,43].

**Figure 6:**
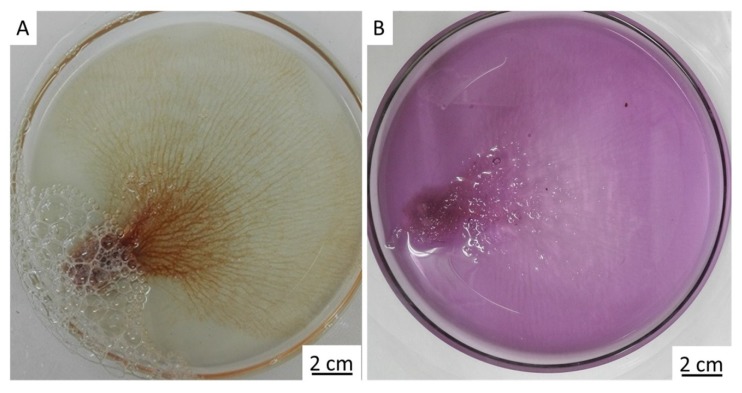
The spherulocite-free chitinous skeleton of *I. flabelliformis* can be isolated after alternating treatment of the construct with acetic acid (**A**) and NaOH (**B**) (see also Figure 7).

**Figure 7:**
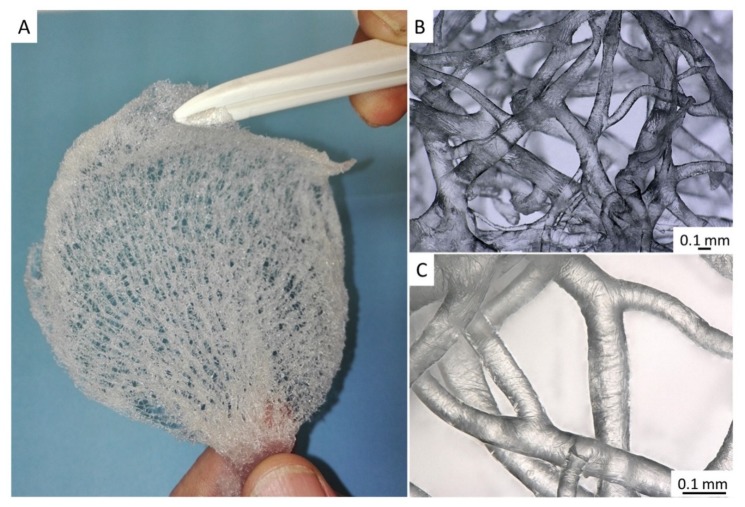
The chitinous skeleton isolated from *I. flabelliformis* (**A**) represents a mechanically elastic, flat, but still three-dimensional (3D)-based construct made of interconnected tubular fibers (**B**). These fibers show excellent capacity for saturation with diverse liquids including water (**C**).

Further treatment of pigmented chitinous fibers using acetic acid and alkali (see Section 4.2) (Figure 6) led to the isolation of ready-to-use 3D chitinous scaffolds which visually resemble the well-known architecture of fabric-based bandage materials (Figure 7).

As reported by us previously [13], similar tubular, flat, 3D scaffolds isolated from the marine demosponge *Ianthella basta* closely related to *I. flabelliformis* [11] were applied in the tissue engineering of hMSCs. However, in this study, we decided to examine the possibility of such constructs as drug delivery matrices for the future development of alternatives to well-recognized antimicrobial textiles (for an overview, see References [44,45,46,47]).

**Figure 8:**
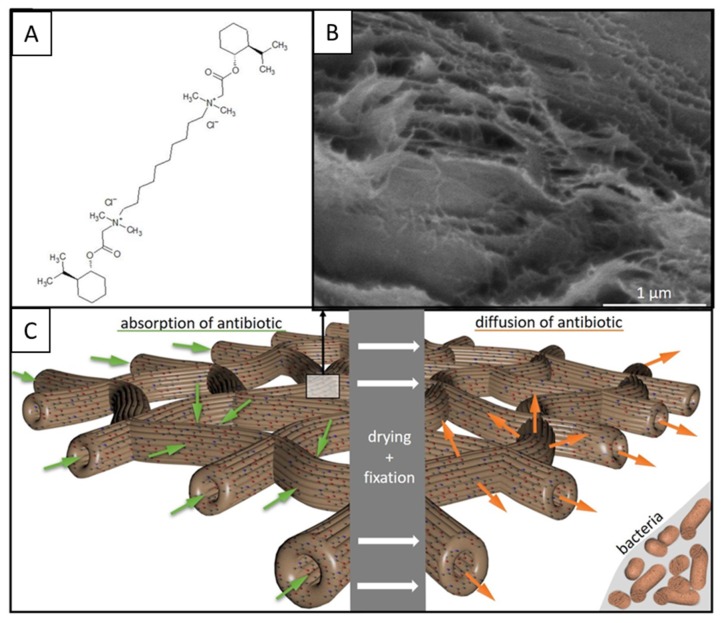
Three-dimensional chitin scaffold as a potential drug delivery matrix. (**A**) Chemical formula of decamethoxine which was used in our study as a model substance with antibiotic activity. The nanoporous structure of *I. flabelliformis* chitin-based tube walls (SEM image, **B**) may be responsible for both the initial absorption of the substance into the organic matrix and for the following diffusion of this substance from the chitinous construct being transferred to the agar plate contaminated with corresponding bacterial cultures (**C**).

We used here the chemical compound 10-[dimethyl-[2-[(1*R*,2*S*,5*R*)-5-methyl-2-propan-2-ylcyclohexyl]oxy-2-oxoethyl]azaniumyl]decyl-dimethyl-[2-[(1*R*,2*S*,5*R*)-5-methyl-2-propan-2-ylcyclohexyl]oxy-2-oxoethyl]azanium;dichloride (Figure 8A), known also as decamethoxine (molecular formula C_38_H_74_Cl_2_N_2_O_4_; molecular weight 693.9 g/mol). It is highly soluble both in water and ethanol. Due to the characteristic nanoporous structure of tubular chitin in verongiid sponges [9,11] (Figure 8B), we suggest that this reagent can easily move through membrane-like walls of chitin fiber both via absorption from the solution and via diffusion (after drying and fixation) on and within the chitinous matrix (Figure 8C). The principal scheme is shown in Figure 8.

**Figure 9:**
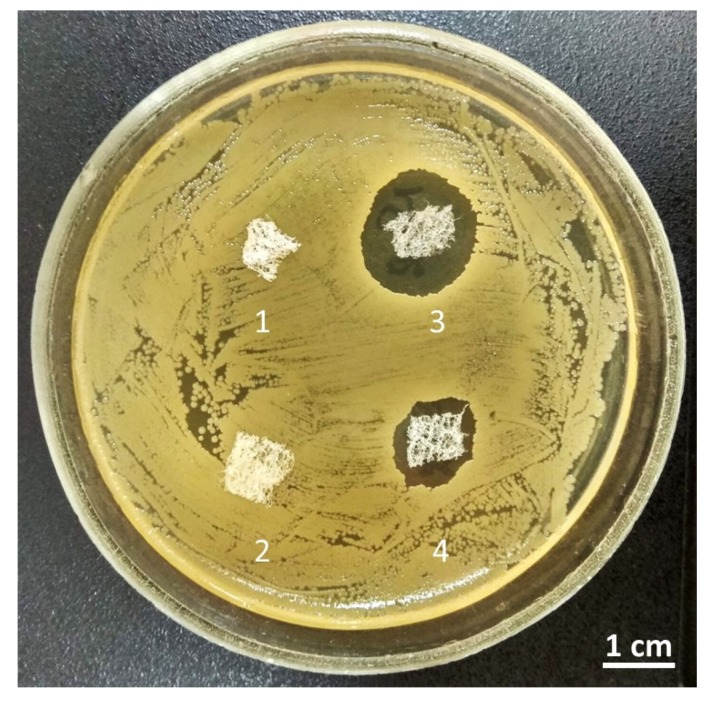
Sponge chitin filled with decamethoxine as an antimicrobial fabric against *Staphylococcus aureus*. 1—Sterile *I. flabelliformis* chitin scaffold (see enlarged image in Figure 7) washed with distilled water as a control sample. 2—Chitin scaffold washed with 70% ethanol and dried as a control sample. 3—Chitin scaffold impregnated with 0.1% water solution of decamethoxine. 4—Chitin scaffold impregnated with 0.1% ethanolic solution of decamethoxin. All observations were carried out after 24 h.

The 3D chitin scaffold of *I. flabelliformis* impregnated with a 0.1% water solution of decamethoxine and dried with sterile filter paper inhibited the growth of *S. aureus* in an agar diffusion assay (6 mm from the edge of the matrix) (Figure 9(3)). The chitin scaffold impregnated with 0.1% ethanol solution of decamethoxine and then dried for ethanol evaporation showed less activity (4-mm growth inhibition from the edge of the matrix) (Figure 9(4)). This phenomenon is based, probably, on the difference between water and ethanol with respect to their hydrophilicity. Both samples retained properties to release antiseptic on their second and third use, after moving to a fresh microbe culture. Control samples of chitinous scaffolds used without any treatment, with the exception of sterilization at 121 °C (Figure 9(1),(2)), did not show any antibacterial activity under the same experimental conditions.

## 3. Discussion

After the discovery of pigmental cells in the fibrous skeletons of Ianthellids by Flemming in 1872, there were numerous reports discussed by de Laubenfels in 1948 where the possible origin, location, and function of these cells was proposed. Carter, for example, described *Ianthella* sponge in 1881 as follows: “*Sarcode charged with dark purple pigmental cells, especially numerous on the surface and in the horny laminae of the fiber, which appear to be secreted by them*.” [34]. At the same time, Lendenfeld (1882) [33] stated the following” “*The most characteristic whereby* Ianthella *is set off is its unique content of fiber cells. These are not scattered through the pith, but are either actually embedded in the fiber walls, or adherent to the inner surface of the hollow cylinder of the fiber which surrounds the pith. These cells must have reached their buried location alive, but how they obtained food and oxygen seems mysterious. Perhaps the pith is quite permeable and acts as supply tube. Another hypothesis is that cells get caught in the forming fiber and die, but leave a pattern of their shape behind, like footprints, or their original substance replaced by bacteria-resistant chemicals.*” [33]. Indeed, until the discovery of chitin in fibrous skeletons of verongiid demosponges by our group in 2007 [8], as well as in the verongiid species of the genus *Ianthella* [11], the most accepted opinion was that skeletal fibers are made of a “horny”, proteinaceous, biomaterial called spongin [6]. It is also well known that pigment oxidation is responsible for the rapid *Ianthella* sponge tissue changes in color (mostly from yellow to purple, or even blackish purple) [33,35,48]. Preserved specimens usually remain in the dark violet condition (Figure 1, Figure 3 and Figure 4A).

In this study, we showed that the pigmental cells of *I. flabelliformis* are located within skeletal fibers between microlayers of chitin (Figure 5) and contain bromotyrosines. Previously, the biosynthesis of several bromotyrosine-related compounds, i.e., bastadins, by *I. flabelliformis* demosponge was reported [41]. Compounds such as bastadin 25, 15-*O*-sulfonatobastadin 11, and bastadin 26 were identified in *I. flabelliformis*; however, their origin was heretofore elusive, with their production in special cells discovered here. Interestingly, there are still no reports of bastadin or araplysillin as typical bromotyrosines of *Ianthella* species being microbially derived [48]. The intriguing question about the possible ancient microbial origin of the pigmental cells remains open.

The biological function of bromotyrosine-producing cells could be based on previously reported results [49] concerning the inhibition of microbial chitinases using bromotyrosines. In this case, verongiids developed a unique chemical defense strategy to protect their skeletal fibrous chitin from bacterial and fungal invasion. Taking into account our discovery of exceptionally preserved chitin in 505-million-years-old fossil remains of the vernogiid sponge *Vauxia gracilenta* [1], we believe that the appearance of this strategy was crucial in the evolution of the sponges belonging to the order Verongiida. Previously, we also showed that partially depigmented chitinous skeletons of selected verongiids are still resistant to diverse bacterial chitinases under experimental conditions in vitro [11]. Only completely purified sponge chitin becomes soluble in chitinase-containing solutions [50,51].

In the near future, we plan to use such a “naturally loaded” bromotyrosine chitin (Figure 3C) to study the possible diffusion of corresponding bromotyrosines using model systems with sea water, physiological solutions, and artificial body fluid.

To our best knowledge, there are no reports on the application of pure chitinous scaffolds for drug delivery. Most papers are dedicated to chitosan or ionically cross-linked chitin microspheres [52]. In one case, a chitin–amphipathic anion/quaternary ammonium salt dressing was prepared [53]. In our study, however, we utilized a recognized antibacterial compound—decamethoxine.

Decamethoxine (its structural formula is shown in Figure 8A) is a cationic gemini surfactant [54], which exhibits strong bactericidal and fungicidal effects. It modifies the permeability of the microbial cell membrane, resulting in the destruction and death of diverse microorganisms [55]. For example, it has a wide spectrum of antimicrobial action on Gram-positive bacteria (*Staphylococcus, Streptococcus, Pneumococcus*), Gram-negative bacteria (*Pseudomonoas, Neisseria gonorrhoeae, Chlamydia trachomatis*) [56], protozoa, dermatophyte, yeast-like fungi of *Candida* genus, and viruses [57]. It was also proven that decamethoxine at a concentration of 10 µg/ml drastically reduces the adhesion of coryneform bacteria, *Salmonella*, *Staphylococcus,* and *Escherichia* [56]. Its method of action may be achieved via adhesion or competitive binding to bacterial adhesins, or to the surface receptors of host cells. Due to its high bacteriostatic effect, decamethoxine is used for the disinfection of surfaces of diverse surgical tools [58], as well as of contact lenses [59].

Our first results (Figure 9) confirmed that decamethoxine can be successfully absorbed from corresponding water and ethanol-containing solutions by chitinous scaffolds isolated from *I. flabelliformis.* Furthermore, this compound can subsequently diffuse from the chitinous matrix surface, as well as, probably, from the inner space of microtubular and nanoporous structures. The appearance of death zones around colonies of *S. aureus* during 24 h of incubation confirms the antibiotic activity of decamethoxine through diffusion from the chitinous scaffold. Now, we need a longer assay including studies on a Fickian diffusion, as well as on possible non-Fickian behavior [60,61], of this previously non-investigated microtubular chitin matrix. On this first stage, we did not differentiate between the release of substance adsorbed to the outside of the matrix, substance absorbed via nanopores, substance sucked up and released via capillary action, etc. Consequently, it is also not clear with what kind of diffusion-controlled system (matrix-type system or reservoir-type system) [62] is used here. Understanding the structure–function relationship of the sponge biomaterial system represented in this study as a new antimicrobial drug release scaffold [63] could be the key to the successful application of this special delivery system. The drug release kinetics [64] with respect to decamethoxine and other antimicrobial compounds which can be used in naturally pre-structured sponge chitin will be studied in detail in the near future.

## 4. Materials and Methods 

### 4.1. Location and Collection

The sponge *Ianthella flabelliformis* (WAM Z87073) was collected by J. Fromont and L. Kirkendale at station SOL47/W/A042 (15°36’46.10” south (S), 124°04’22.92” east (E) to 15°36’44.77” S, 124°04’22.38” E), Kimberley, Western Australia in March 2015 at a depth of 35.3–35.5 m. Morphological identification was supported by molecular barcoding and comparison against reference materials of *I. flabelliformis* and other *Ianthella* spp. from the Western Australian Museum using the 28S ribosomal RNA (rRNA) C-region barcoding region for sponges (see Reference [65] for methodological details).

### 4.2. Isolation of Chitinous Skeleton from the Sponge and Identification of Selected Bromotyrosines

The isolation of chitinous scaffolds from the ianthellid sponges was conducted as described by us previously [11]. In brief, it was performed in three main steps: (i) sponge skeletons were washed three times with distilled water for the removal of water-soluble compounds, and then bromotyrosines were extracted with methanol; (ii) residual fragments were treated with NaOH (2.5 M, Merck) at 37 °C for 72 h for deproteinization; (iii) lastly, the isolated scaffolds were treated with acetic acid (20%, Roth) at 37 °C for a period of 6 h to remove residual calcium and magnesium carbonates, and then washed in distilled water up to pH 6.8. This isolation procedure was repeated three times to obtain colorless tubular scaffolds. The purity of isolated chitinous scaffolds was proven using standard analytical procedures as described previously [11].

The methanolic extracts of sponge fragments shown in Figure 3C were analyzed using a Shimadzu HPLC system, coupled to an ultraviolet–visible light (UV–Vis) detector (Shimadzu, Kyoto, Japan; Waters SunFire Prep OBD C18 column (30 × 75 mm)). Routine detection was at 215 and 241 nm. A solvent system consisting of MeCN (A) and H_2_O (B) at a gradient increasing linearly from 0% to 100% was used for compound separation. LCMS analyses were carried out on an Agilent 1100 (Agilent, Santa Clara, California, USA) LC system equipped with a G1956 MSD detector. A Zorbax C18 RR column was used, and gradient elution with 0.1% HCOOH in H_2_O–MeCN was applied. 

### 4.3. Antimicrobial Activity of Chitin Matrix

The prepared chitin scaffold of *I. flabelliformis* was cut into 1-cm^2^ squares, washed twice for 15 min in sterile distilled water, and put into 0.1% (*w*/*v*) water or ethanol solution of decamethoxine (Yuria-pharm, Kyiv, Ukraine). Samples used for control were put into sterile distilled water or 70% ethanol. After 2 h of incubation, the samples were dried with sterile filter paper (for the water solution) or in a thermostat (for ethanol-based solutions). Dry samples were placed on a Petri dish with fresh culture of a clinical strain of *Staphylococcus aureus* ATCC 6538P (FDA 209P) on meat–peptone agar (MPA) and cultivated for 24 h at 37 °C. In 24 h, the zones of growth inhibition for first use were measured, and samples were moved with sterile forceps to a Petri dish with a fresh daily culture of *S. aureus.* Cultivation and measuring were repeated three times with the same samples of chitin scaffold and fresh cultures (first use, second use, and third use). All tests were provided with proper control (sterility control of nutritive environment (MPA), and control of microorganism growth without compound).

### 4.4. Stereomicroscopy and Light Microscopy Imaging

*I. flabelliformis* sponge samples in different stages of chemical treatment and isolated chitinous scaffolds were observed with a Keyence VHX-6000 (Keyence, Osaka, Japan) digital optical stereomicroscope, and using a BZ-9000 microscope (Keyence, Osaka, Japan) in the light microscopy mode (Machalowski et al., 2019).

### 4.5. Transmission Electron Microscopy (TEM), Scanning Electron Microscopy (SEM), and EDX

For TEM investigations, samples of *I. flabelliformis* chitin as represented in Figure 3C were fixed with 2.5% glutaraldehyde in phosphate-buffered saline (PBS) at room temperature, post-fixed with 1.5% osmium tetroxide, dehydrated in a graduated series of acetone (including a staining step with 1% uranylacetate), and embedded in Epoxy resin according to Spurr (1969) [66]. Ultra-thin sections (about 70 nm) of samples were prepared on a Leica EM UC6 ultramicrotome (Leica, Wetzlar, Germany) equipped with a Diatome diamond knife, mounted on Pioloform-coated copper grids, post-stained with uranylacetate and lead citrate (according to Reynolds, 1963 [67]), and analyzed using a Zeiss CTEM 902 (Carl Zeiss, Wetzlar, Germany) at 80 kV (University of Bayreuth).

For SEM analysis, samples were prepared as described for TEM analyses without osmium tetroxide and uranylacetate. For elemental analyses (EDX), the block faces of samples were cut on a Leica EM UC6 ultramicrotome equipped with a Diatome diamond knife, carbon-coated, mounted on an SEM sample holder, and analyzed on a Philips ESEM XL 30 (FEI Company, Peabody, MA, USA) at suitable accelerating voltages. For EDX spectra, accelerating voltages between 15 kV and 30 kV were used. 

## 5. Conclusions 

**Figure 10:**
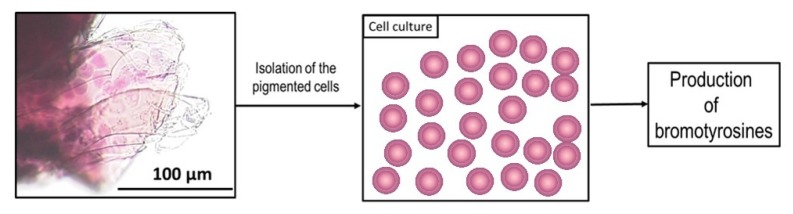
Schematic overview of the challenging tasks: in the near future, we must elaborate an effective method for the isolation of pigmental, bromotyrosine-producing cells from chitinous skeletal fibers of ianthellids with the aim of obtaining cell cultures which should be able to synthetize corresponding bromotyrosines using bioreactors.

The demand of biomaterials of natural origin is increasing for different reasons, including the major sustainability in their production pipeline leading to the reduction of environmental impact of microplastics and of CO_2_ emissions. In this context, the chitin-based materials of marine origin are considered of recent interest, and the results of this work clearly underline that sponges of the order Verongiida can now be considered as a relevant resource in this perspective. The methodology here developed for the first time on *I. flabelliformis* allows a double exploitation of this sponge species: (i) as a source of bromotyrosines of well-known pharmaceutical properties, and (ii) as a source of unique biomaterial that could potentially substitute artificial fabric-based bandages, for its peculiar structural organization and for its capacity to be filled with anti-bacterial compounds. The purified 3D chitin matrix isolated from *I. flabelliformis* does not have innate antibacterial activity, but is prospective as a naturally prefabricated dressing material due to its ability for incorporation of antiseptic solutions using the capillary effect or the fixing of dry antiseptic on the surface of its nanoporous, membrane-like chitin microtubes. Consequently, the very promising results here shown are prodromal for further development both in the field of mariculture and of sponge cell biology. The production of chitin-based scaffolds will be in fact realistically sustainable only after a careful analysis of the farming potentialities of this sponge species. Its widespread presence in the Indo-Pacific does not exclude “a priori” the possibility to develop sponge farming facilities in many geographical areas, and the concrete feasibility of this approach is the consequential evolution of this study. The bromotyrosines produced by *I. flabelliformis* are also well-known interesting compounds. On this specific topic, the interest will be to improve their production not only via simple extraction from farmed sponges but also by developing specific bioreactors for culturing the bromotyrosine-producing cells isolated from the sponge chitinous structures (Figure 10).

## Supplementary Materials

The following are available online at https://www.mdpi.com/1660-3397/17/10/574/s1: Figure S1: Structures of bromotyrosines isolated from *l. flabelliformis*, Figure S2: LCMS spectrum of isolated compound (Bastadin 25), Figure S3: LCMS spectrum of isolated compound (Araplysillin-I N20-sulfamate).
