# Supplementary material for: Naturally Drug-Loaded Chitin: Isolation and Applications
Source: Mar Drugs. 2019 Oct 10;17(10):574. doi: 10.3390/md17100574 (PMC6835269; doi:10.3390/md17100574)
Supplement: Supplementary file 1 [file marinedrugs-17-00574-s001.pdf]

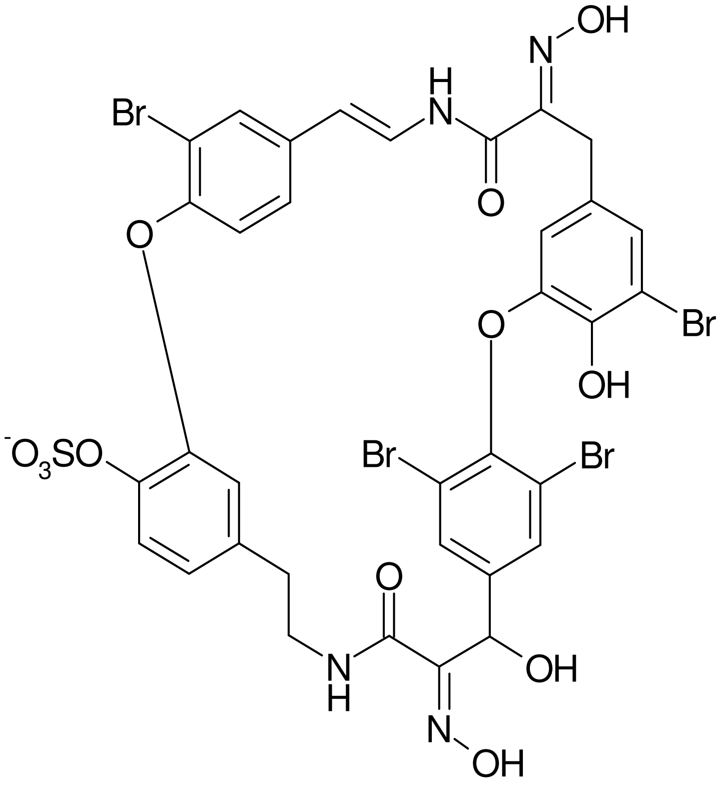

Bastadin 25

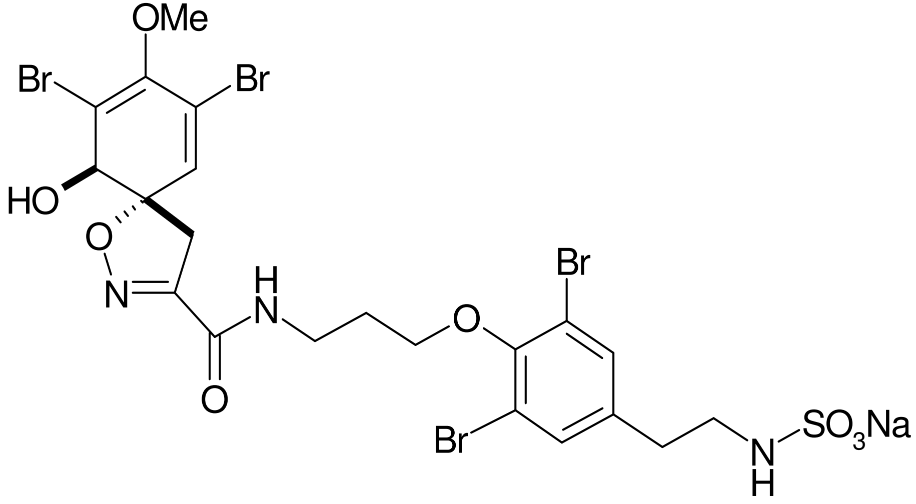

Araplysillin-I N^{20}-sulfamate

Figure S1. Structures of bromotyrosines isolated from *l. flabelliformis*

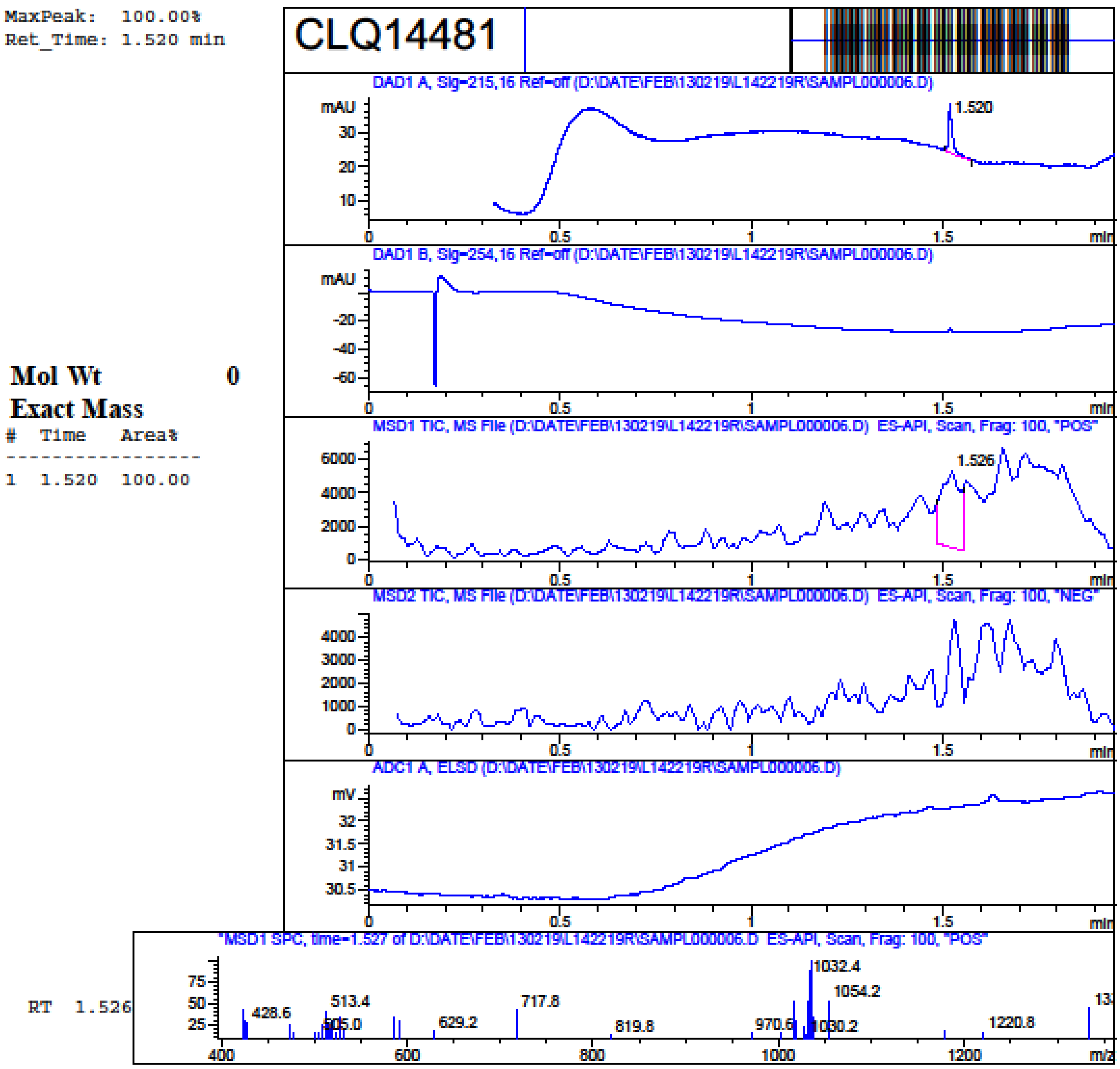

Figure S2. LCMS spectrum of isolated compound (Bastadin 25)

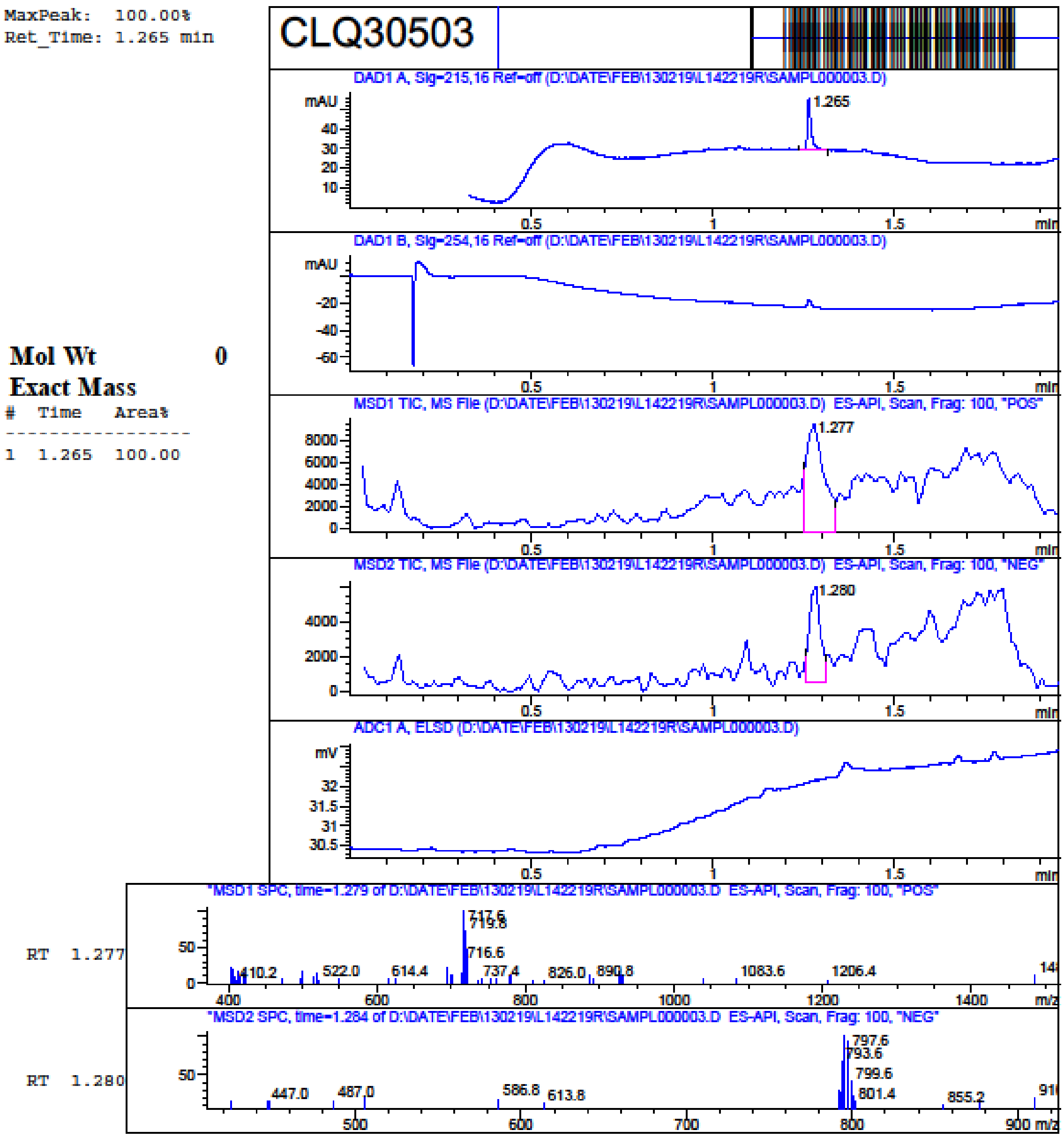

Figure S3. LCMS spectrum of isolated compound (Araplysillin-I N^{20}-sulfamate)
